# *Corynebacterium striatum* ‐Associated Pyogenic Osteomyelitis With Direct Extension From Postoperative Empyema

**DOI:** 10.1002/rcr2.70230

**Published:** 2025-06-04

**Authors:** Shinnosuke Fukushima, Kazuhiro Noma, Hideharu Hagiya

**Affiliations:** ^1^ Department of Infectious Diseases Okayama University Hospital Okayama Japan; ^2^ Department of Gastroenterological Surgery Okayama University Graduate School of Medicine, Dentistry and Pharmaceutical Sciences Okayama Japan

**Keywords:** *Corynebacterium striatum*, empyema, osteomyelitis, rifampicin

## Abstract

*Corynebacterium striatum* can cause postoperative empyema. *C. striatum* ‐associated empyema may be associated with osteomyelitis. Rifampicin is a viable therapeutic option for *C. striatum* infection.

## Graphical abstract

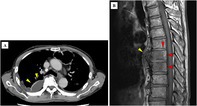

A 72‐year‐old Japanese man was admitted to our hospital for surgery for oesophageal cancer. On postoperative day 13, thoracentesis was performed for pleural effusion (Figure 1A). Microbiological culture yielded a monomicrobial isolation of *Corynebacterium striatum* , for which vancomycin was initiated under the diagnosis of postoperative empyema. Two weeks later, the patient developed back pain, and magnetic resonance imaging revealed acute osteomyelitis that radiologically localised to the anterior region of Th 8 and 9 vertebrae with T1‐weighted image (Figure 1B), suggesting a direct extension of the inflammatory process from the empyema. Blood cultures were negative throughout the postoperative course. The antibiotic susceptibility test for the *C. striatum* isolate showed susceptibility to vancomycin, trimethoprim‐sulfamethoxazole, and rifampicin (RFP). Following 3 weeks of intravenous vancomycin treatment with a favourable clinical course, the therapeutic regimen was switched to oral administration of RFP and sulfamethoxazole‐trimethoprim combination based on the antibiotic susceptibility test, and the patient was discharged. The antibiotic therapy continued for a total of 6 weeks without recurrence.

**FIGURE 1:**
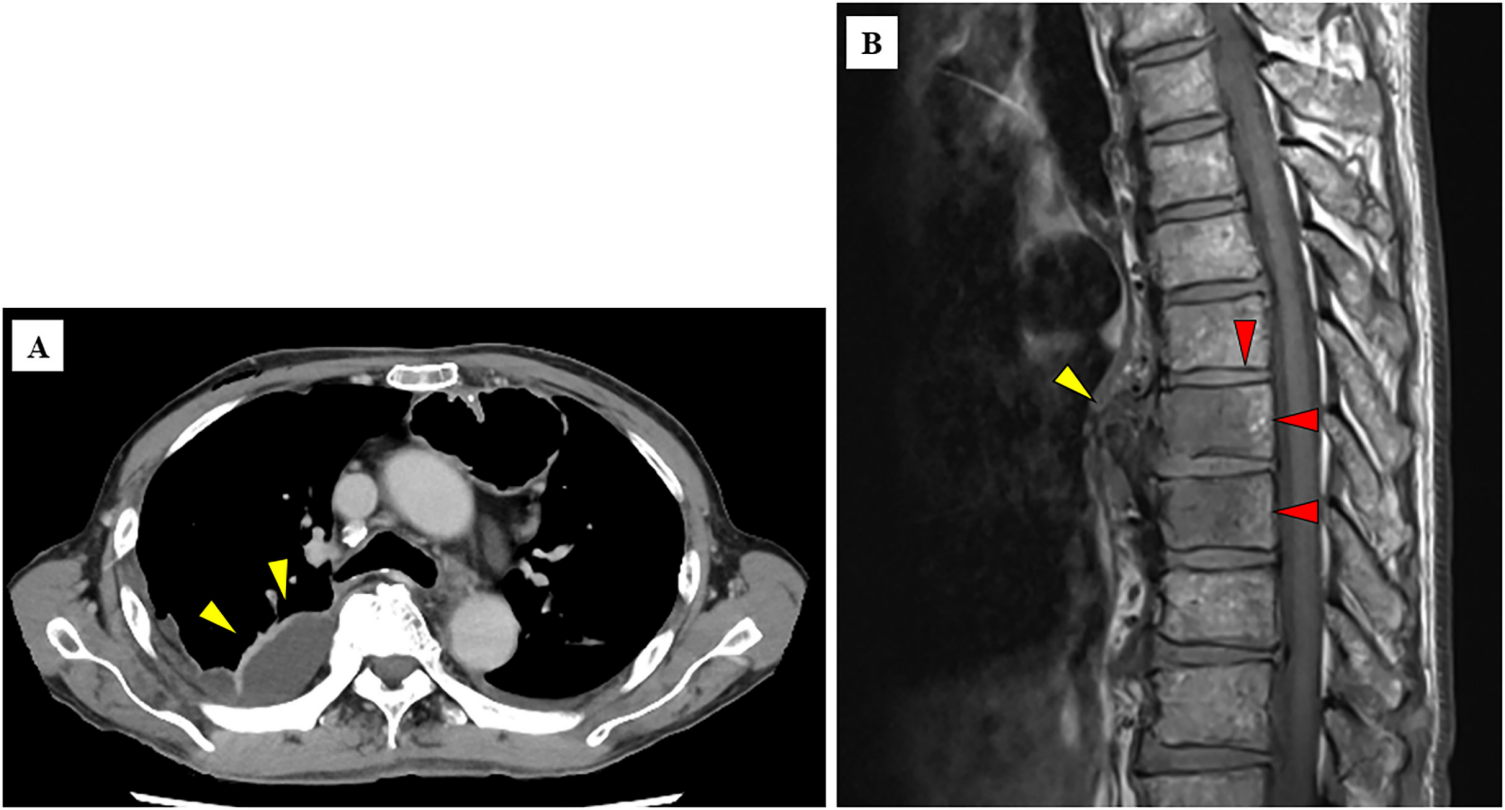
(A) Contract‐enhanced computed tomography (CT). (B) Magnetic resonance imaging (MRI). (A) Chest contract‐enhanced CT shows a right pleural effusion with a contrast‐enhancing membrane (yellow arrows). (B) MRI with T1‐weighted shows low signal localised to the anterior region of the Th 8 and 9 vertebrae body (red arrows) through osteophytes on the front of the vertebrae (yellow arrow), preserving intervertebral discs.

This case highlights that empyema caused by *C. striatum* potentially develops secondary vertebral osteomyelitis. *C. striatum* can cause pneumonia and empyema, while it is rarely associated with osteomyelitis [1]. Although the organism demonstrates complete susceptibility to vancomycin, switching to oral therapy including RFP has been reported because the sensitivity to RFP is retained [2]. *C. striatum* can induce postoperative empyema, and RFP represents a viable therapeutic option.

## Author Contributions

S.F. drafted and H.H. revised the manuscript; K.N. managed the patient; all authors gave final approval to the submitted manuscript.

## Ethics Statement

Written informed consent was obtained from the patient for the publication.

## Conflicts of Interest

The authors declare no conflicts of interest.

## Data Availability

Data sharing not applicable to this article as no datasets were generated or analysed during the current study.
